# Association of Type D Personality with Disability and Quality of Life in Patients with Chronic Nonspecific Low Back Pain

**DOI:** 10.3390/healthcare14111439

**Published:** 2026-05-22

**Authors:** Esra Şahingöz Bakırcı, Muhammed Balcı, Tuğba Alışık

**Affiliations:** 1 Department of Physical Medicine and Rehabilitation, Faculty of Medicine, Yozgat Bozok University, 66000 Yozgat, Türkiye; dresrasahingoz@gmail.com; 2 Department of Physical Medicine and Rehabilitation, Faculty of Medicine, AIBU İzzet Baysal Physical Treatment and Rehabilitation Training and Research Hospital, Bolu Abant İzzet Baysal University, 14030 Bolu, Türkiye; mbalci.dr@gmail.com

**Keywords:** low back pain, chronic, type D personality, self concept, disability evaluation, quality of life

## Abstract

Background and Objectives: This research aimed to compare Type D personality characteristics and self-esteem between individuals with chronic NSLBP and healthy controls, while also exploring their relationships with functional status, psychological symptoms, and quality of life within the patient group. Materials and Methods: In this cross-sectional investigation, 34 patients with chronic NSLBP were compared with 34 healthy controls with similar age and sex distribution. Pain intensity was quantified via the Visual Analog Scale (VAS), while functional impairment was evaluated using the Oswestry Disability Index (ODI). Psychological profiling included the Type D Scale-14 (DS14) for personality traits, the Rosenberg Self-Esteem Scale (RSES) for self-worth, and the Hospital Anxiety and Depression Scale (HADS) for emotional distress. Health-related quality of life was captured through the 12-Item Short Form Health Survey (SF-12). Results: Type D personality was significantly more prevalent in the NSLBP group than in controls (50% vs. 20.6%, *p* = 0.011). Patients with NSLBP had significantly higher negative affectivity (NA) scores (*p* < 0.001) and anxiety scores (*p* = 0.007) and lower SF-12 Physical Component Summary scores (*p* < 0.001) than controls. Pain intensity and disability were positively correlated with Type D personality traits, particularly NA and the Type D composite score. In exploratory subgroup analyses, patients with Type D personality also had higher pain intensity, disability, anxiety, and depression scores and lower SF-12 Mental Component Summary (MCS) scores than those without Type D personality. In adjusted regression analyses within the NSLBP group, Type D personality was associated with higher VAS (*p* = 0.004) and ODI scores (*p* = 0.007) and lower SF-12 MCS scores (*p* = 0.003). Conclusions: Type D personality characteristics were more frequent in patients with chronic NSLBP than in healthy controls and were associated with higher pain intensity, greater disability, higher anxiety and depressive symptom scores, and poorer mental quality-of-life scores within the patient group. In contrast, self-esteem did not differ significantly between patients and controls. Due to the inherent constraints of a cross-sectional framework and the potential construct redundancy between NA and emotional distress, the current results signify correlational links rather than definitive causality. Consequently, subsequent prospective research is vital to delineate the temporal dynamics and the long-term predictive value of Type D personality traits in the progression of chronic NSLBP.

## 1. Introduction

Epidemiological data consistently identify low back pain as a major clinical problem within modern healthcare, maintaining its position as a primary global driver of protracted functional impairment [1]. In most patients, low back pain cannot be attributed to a distinct structural abnormality or identifiable pathological condition; such cases are therefore categorized as nonspecific low back pain (NSLBP) [2,3]. Pain lasting longer than 12 weeks is considered chronic, and patients frequently experience limitations in daily functioning along with a decline in quality of life [4]. Due to its frequent occurrence and persistent course, chronic NSLBP represents a substantial burden for patients and healthcare systems.

Current evidence supports a biopsychosocial framework for NSLBP, suggesting that clinical outcomes are influenced by complex interactions between biological markers and psychological determinants [3]. In chronic NSLBP, the degree of functional impairment frequently appears disproportionate to pain severity alone, suggesting an important contribution of psychological factors [5]. Psychosocial factors linked to an unfavorable prognosis in low back pain are commonly termed “yellow flags.” Pain chronicity and increased disability have been linked to negative pain-related beliefs, fear-avoidance behaviors, depressive symptoms, and passive coping strategies. The present results indicate that clinical outcomes may be influenced not only by pain severity itself but also by the way individuals perceive and cognitively process their pain experience [6]. However, many of these psychosocial factors are state-related variables that may change over time. In contrast, stable personality traits may represent relatively enduring psychosocial factors associated with the chronic pain experience. Within this framework, personality traits may represent an important psychosocial factor related to the experience and clinical impact of chronic pain.

Type D personality is defined by the combination of negative affectivity (NA) and social inhibition (SI). NA refers to a predisposition toward experiencing emotions such as anxiety, irritability, and sadness, whereas SI describes the tendency to avoid expressing emotions in social situations because of fear of disapproval or negative evaluation by others. Individuals with this personality profile may experience negative emotions more intensely, yet often avoid expressing them in interpersonal situations [7]. Type D personality has previously been recognized as a relevant psychological factor in various clinical populations. Notably, this personality profile was first recognized for its strong association with adverse clinical outcomes in patients with cardiovascular diseases [8]. In individuals with hypothyroidism, it has been associated with dissatisfaction with treatment and care [9]. In patients undergoing spinal surgery, Type D personality traits have been associated with greater postoperative pain and delayed recovery [10]. Type D personality characteristics have been observed more frequently in patients with fibromyalgia than among healthy populations. Moreover, the presence of this personality profile appears to correlate with intensified symptom severity in this population [11]. These findings suggest that personality characteristics may influence both pain perception and treatment outcomes. Despite several reports describing differences in personality profiles between individuals with chronic NSLBP and healthy individuals, data specifically examining personality characteristics in this patient group are still scarce. Although the association of Type D personality with various chronic diseases has been widely investigated, its potential role in the clinical burden of chronic NSLBP remains insufficiently studied [5,10,12,13].

Self-esteem is an important psychosocial factor, representing an individual’s subjective evaluation of their own value. In the context of chronic illness, this sense of self-worth is often shaped by variables such as social support networks, functional independence, and overall clinical prognosis. Within the specific domain of chronic musculoskeletal pain, evidence indicates a notable link between self-esteem levels and the perceived severity of pain [14]. Reduced self-esteem has been reported more frequently in patients with fibromyalgia than in healthy individuals [15]. In addition, perceived stigmatization and diminished self-esteem are frequently observed in individuals with chronic pain, and these psychosocial factors may be closely linked to the way pain is experienced [16]. These findings further support the concept that chronic pain should be considered a multidimensional condition involving substantial psychosocial influences rather than merely a physical disorder. Although Type D personality has previously been investigated in conditions such as fibromyalgia, myofascial pain syndrome, osteoarthritis, and spine outpatient populations, evidence specifically addressing chronic NSLBP remains scarce. In addition, only a limited number of studies have examined Type D personality and self-esteem together within a comprehensive framework including pain severity, disability, psychological symptoms, and quality of life.

Accordingly, this study aimed to investigate the frequency of Type D personality characteristics and self-esteem levels in individuals with chronic NSLBP compared with healthy controls. Additionally, we sought to investigate the relationships of these psychosocial factors with pain intensity, functional impairment, psychological distress, and quality of life among individuals with chronic NSLBP. We hypothesized that individuals with chronic NSLBP would exhibit a higher prevalence of Type D personality characteristics and greater psychological distress compared with healthy controls. We also hypothesized that, among individuals with NSLBP, Type D personality characteristics would be related to more severe pain, higher disability levels, and lower quality of life. Given the limited evidence regarding self-esteem in chronic NSLBP, analyses involving self-esteem were considered exploratory.

## 2. Materials and Methods

This cross-sectional study was performed at the Izzet Baysal Physical Therapy and Rehabilitation Training and Research Hospital affiliated with Abant İzzet Baysal University. Patients were recruited from the Physical Medicine and Rehabilitation outpatient clinics during the period between October 2024 and October 2025.

Participants included individuals aged 18–50 years who had experienced chronic nonspecific low back pain for at least three months. To limit the impact of degenerative changes associated with aging and accompanying comorbidities, the study population was restricted to individuals younger than 50 years. The clinical evaluation included detailed history taking, assessment of red flags, neurological examination, and screening for radicular symptoms. Imaging findings were reviewed when available or when clinically indicated; routine imaging was not required for all participants. Specific causes of low back pain, including significant disc herniation, spinal stenosis, inflammatory, infectious, neoplastic, traumatic, and systemic causes, were excluded based on clinical evaluation and imaging findings when indicated. Significant disc herniation was defined as disc pathology associated with radicular symptoms, neurological deficits, or clinically relevant nerve root compression. Enrollment was conducted on a consecutive basis for all eligible patients who provided informed consent. Healthy volunteers without a history of chronic spinal pain, musculoskeletal conditions requiring medical care, or ongoing pain complaints were recruited as the control group. They were recruited consecutively during the same study period from hospital staff and volunteers accompanying patients attending the outpatient clinic. Controls were selected to have a similar age and sex distribution to the patient group, and no individual matching was performed.

To achieve a more homogeneous study population, patients with a specific cause of low back pain, chronic inflammatory diseases, chronic hepatic or renal insufficiency, active infection, or hepatitis were not included. In addition, individuals with a self-reported physician-diagnosed psychiatric disorder or regular psychiatric treatment were excluded. The Hospital Anxiety and Depression Scale (HADS) was administered to evaluate current levels of anxiety and depressive symptoms, rather than for diagnostic psychiatric assessment. Participants unable to understand the study instructions or complete the questionnaires independently were excluded because of cognitive impairment interfering with participation. Cognitive status was evaluated clinically by the investigator according to each participant’s ability to comprehend the study procedures and complete the questionnaires without assistance. Pregnant and breastfeeding women were also not included in the study.

Type D personality characteristics were evaluated using the Turkish version of the Type D Scale-14 (DS14), which was originally developed by Denollet and includes two subdimensions: NA and SI. NA reflects a predisposition toward experiencing emotions such as anxiety, irritability, and sadness, whereas SI describes the tendency to avoid emotional expression and social interaction because of concerns about rejection or negative evaluation. Both subdomains contain seven items scored on a five-point Likert scale ranging from 0 to 4. In accordance with established cutoff criteria, individuals with scores of 10 or higher on both the NA and SI dimensions were categorized as having Type D personality. Furthermore, we computed a composite interaction score (NA × SI) for further analysis [17,18]. Type D personality status based on DS14 cut-off scores was considered the primary Type D-related variable. NA, SI, and the NA × SI composite score were analyzed as secondary exploratory dimensional indicators.

Sociodemographic data, comorbid conditions, pain duration, and pain severity were documented for all participants. Current low back pain intensity at the time of assessment was evaluated using a 100 mm Visual Analog Scale (VAS), ranging from “no pain” (0 mm) to “worst imaginable pain” (100 mm). Participants indicated their perceived pain level by placing a vertical mark on the line; the distance from the origin to this point was then measured in millimeters to derive the final VAS score. In this scoring system, elevated values represent more severe pain intensity [19].

The Turkish-validated version of the Oswestry Disability Index (ODI) was employed to evaluate low back pain-related disability. This instrument evaluates limitations in daily activities across ten domains, including basic physical functions such as lifting, walking, standing, and sitting, as well as aspects of daily life such as personal care, sleep, social participation, sexual activity, and travel. Each item is rated on a scale from 0 to 5, and higher total scores reflect greater functional limitation [20,21].

The Rosenberg Self-Esteem Scale (RSES) was utilized to measure the global self-worth of the study population. This instrument comprises 10 items, each scored on a four-point Likert-type scale ranging from 0 to 3. To ensure accurate assessment, negatively framed items were reverse-coded prior to analysis. The cumulative score ranges from 0 to 30, where elevated scores represent a more robust sense of self-esteem [22]. The Turkish adaptation of the RSES has previously established its psychometric robustness in terms of validity and reliability [23].

Psychological distress was evaluated via the HADS, a 14-item self-assessment tool. The scale is bifurcated into two distinct domains: anxiety (HADS-A) and depression (HADS-D), with seven items dedicated to each. Scores for each subscale range from 0 to 21, where an upward trend in scoring reflects increased symptom severity [24]. Previous methodological research has confirmed the internal consistency and structural validity of the scale’s Turkish version [25].

12-Item Short Form Health Survey (SF-12) was administered to measure the health-related quality of life of the participants. The questionnaire yields two summary measures: the SF-12 Physical Component Summary (SF-12 PCS) and the SF-12 Mental Component Summary (SF-12 MCS). The SF-12 PCS reflects domains related to physical functioning, role limitations, bodily pain, and general health perception, whereas the SF-12 MCS evaluates vitality, social functioning, and emotional well-being. Scores for both components range from 0 to 100, with higher scores indicating better health-related quality of life [26,27].

The study protocol was reviewed and formally approved by the Abant Izzet Baysal University Clinical Research Ethics Committee (Decision No: 2023/302; Date: 10 October 2023). All study procedures were carried out in accordance with the ethical standards outlined in the Declaration of Helsinki. Each participant was thoroughly informed regarding the study’s aims and procedures, and subsequently provided both written and verbal informed consent prior to their inclusion in the study.

Because no prior study specifically examining both Type D personality and self-esteem in patients with chronic nonspecific low back pain was available, the sample size calculation was derived from the most methodologically comparable study identified in the literature. An a priori power analysis was performed using G*Power software version 3.1.9.7 (Heinrich-Heine-Universität Düsseldorf, Düsseldorf, Germany). Based on previously reported Type D personality frequencies in patient and control groups (58.6% and 21.7%, respectively), with a two-sided significance level of 0.05, 80% power, and equal group allocation, the estimated minimum sample size was 31 participants for each group (total *n* = 62) according to Fisher’s exact test for two independent proportions.

### Statistical Analysis

Data analyses were conducted using JASP version 0.95.4 and jamovi version 2.7.9. Normality of continuous variables was examined through the Shapiro–Wilk test together with histogram and Q–Q plot inspections. Variance homogeneity was evaluated using Levene’s test. No missing values were identified among the analyzed variables. Potential outliers were examined using boxplot distributions and standardized residual values, and suspicious observations were checked against the original records. Continuous data were expressed as mean ± standard deviation or median with interquartile range according to data distribution, whereas categorical variables were reported as numbers and percentages. Comparisons between groups were conducted using Student’s *t*-test or Mann–Whitney U test for continuous variables and chi-square or Fisher’s exact test for categorical variables when appropriate. Effect size estimates were presented as Cohen’s d for parametric comparisons, rank-biserial correlation with 95% confidence intervals for Mann–Whitney U analyses, and phi coefficient or Cramér’s V for categorical analyses according to table size.

Type D personality status, defined according to the established DS14 cut-off criteria, was considered the primary Type D-related variable. The NA and SI subscale scores and the NA × SI composite score were analyzed as secondary exploratory dimensional indicators. Spearman correlation analysis was used to examine associations among clinical and psychosocial variables. Within-patient comparisons according to Type D personality status and correlation analyses were considered exploratory. Given the number of comparisons and correlations, these findings were interpreted cautiously.

Binary logistic regression analysis was performed to evaluate the association between Type D personality and group status after adjustment for age, sex, and BMI. In addition, multivariable linear regression analyses were conducted within the NSLBP group to examine the relationships between Type D personality and VAS, ODI, RSES, SF-12 PCS, and SF-12 MCS scores while controlling for age, sex, BMI, and pain duration. Considering the relatively small sample size, the number of covariates included in the multivariable analyses was kept limited to clinically meaningful variables in order to minimize model overfitting. HADS-A and HADS-D scores were not entered into the primary adjusted models because of their conceptual and statistical overlap with the negative affectivity dimension of Type D personality. Model assumptions were evaluated by inspection of residual plots, assessment of multicollinearity using variance inflation factors, and examination of influential observations. A two-sided *p* value below 0.05 was accepted as statistically significant.

## 3. Results

The study sample consisted of 34 individuals with chronic NSLBP and 34 healthy controls. The groups were similar in terms of age, sex distribution, BMI, marital status, and smoking status (Table 1). Regarding comorbid conditions, the NSLBP group exhibited a higher numerical frequency; however, these findings did not achieve statistical significance (*p* > 0.05 for all).

Patient-specific clinical characteristics and between-group comparisons of psychosocial and quality-of-life measures are presented in Table 2. In the NSLBP group, the mean VAS score was 7.0 ± 2.0, the median pain duration was 24 (12–81) months, and the median ODI score was 24.5 (18.5–35.5). No significant difference in RSES scores was observed between the NSLBP and control groups (22.1 ± 4.5 vs. 23.3 ± 4.6, *p* = 0.269). NA subscale scores were significantly elevated in the NSLBP group (*p* < 0.001), while no statistically significant between-group difference was identified for SI subscale scores (*p* = 0.263). The NA × SI composite score was also significantly higher in the NSLBP group (*p* = 0.012). The NSLBP group showed elevated HADS-A scores compared with controls (*p* = 0.007), whereas HADS-D scores were comparable between groups (*p* = 0.635). SF-12 PCS scores were reduced in the NSLBP group compared with controls (*p* < 0.001), whereas SF-12 MCS scores were comparable between the groups (*p* = 0.415).

**Figure 1:**
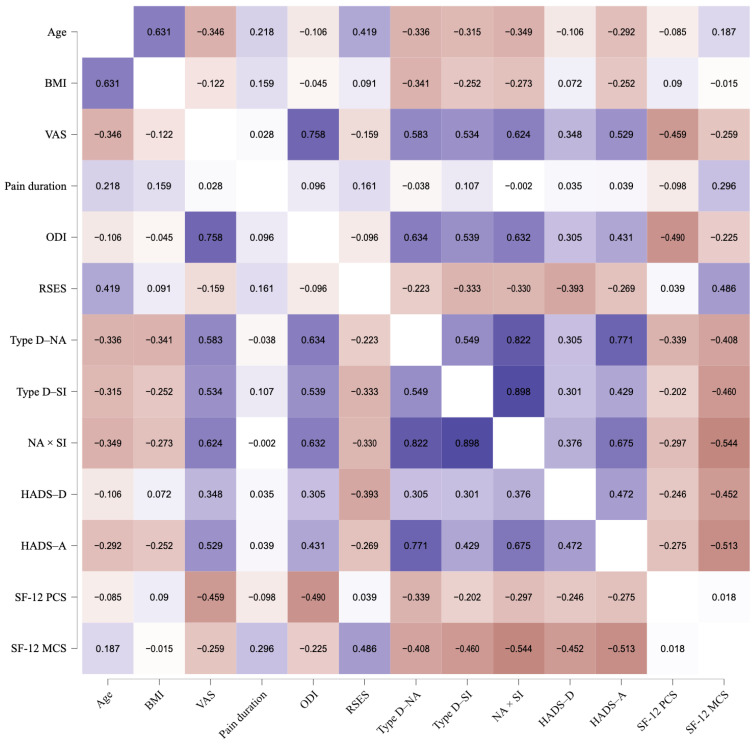
Exploratory Spearman correlation matrix of clinical and psychosocial variables. Values in the cells represent Spearman’s correlation coefficients. The color gradient represents the direction and magnitude of the correlation coefficients; different colors indicate the direction of the correlation, whereas the color intensity reflects its magnitude. Sample size varied by variable pair: correlations involving VAS, ODI, or pain duration were based on the chronic NSLBP group only (*n* = 34), while correlations among variables available for both groups were based on the full study sample (*n* = 68). BMI: body mass index; VAS: Visual Analog Scale; ODI: Oswestry Disability Index; RSES: Rosenberg Self-Esteem Scale; NA: negative affectivity; SI: social inhibition; NA × SI: Type D composite score; HADS-D: Hospital Anxiety and Depression Scale-Depression; HADS-A: Hospital Anxiety and Depression Scale-Anxiety; SF-12 PCS: 12-Item Short Form Physical Component Summary; SF-12 MCS: 12-Item Short Form Mental Component Summary, *n*: number of participants. Correlation analyses were exploratory and were interpreted cautiously because of multiple testing.

Exploratory Spearman correlation analysis demonstrated a strong positive association between VAS and ODI scores (r = 0.758, *p* < 0.001). VAS scores were also positively correlated with Type D NA (r = 0.583, *p* < 0.001), SI (r = 0.534, *p* < 0.01), the NA × SI composite score (r = 0.624, *p* < 0.001), and HADS-A scores (r = 0.529, *p* < 0.01). Similarly, ODI scores were positively correlated with Type D NA (r = 0.634, *p* < 0.001), SI (r = 0.539, *p* < 0.01), the NA × SI composite score (r = 0.632, *p* < 0.001), and HADS-A scores (r = 0.431, *p* < 0.01). RSES scores were negatively correlated with Type D NA (r = −0.414, *p* < 0.001), SI (r = −0.390, *p* < 0.01), the NA × SI composite score (r = −0.453, *p* < 0.001), HADS-D (r = −0.402, *p* < 0.001), and HADS-A (r = −0.397, *p* < 0.001). SF-12 PCS scores were negatively correlated with VAS (r = −0.459, *p* < 0.01), ODI (r = −0.490, *p* < 0.01), and the NA × SI composite score (r = −0.414, *p* < 0.001). SF-12 MCS scores were negatively correlated with Type D NA (r = −0.448, *p* < 0.001), the NA × SI composite score (r = −0.471, *p* < 0.001), and HADS-A (r = −0.454, *p* < 0.001). These exploratory correlations are presented in Figure 1 and should be interpreted cautiously because of multiple testing.

Patients with chronic NSLBP were further evaluated according to Type D personality status as an exploratory subgroup analysis (Table 3). Among patients with chronic NSLBP, those with Type D personality demonstrated higher VAS and ODI scores compared with patients without Type D personality (*p* = 0.003 and *p* = 0.014, respectively). Among patients with Type D personality, both HADS-A and HADS-D scores were elevated compared with those without Type D personality (*p* < 0.001 and *p* = 0.025, respectively), while SF-12 MCS scores were lower (*p* = 0.002). No significant differences were identified between Type D-positive and Type D-negative patients with respect to age, BMI, pain duration, RSES scores, or SF-12 PCS scores.

After adjustment for age, sex, and BMI, binary logistic regression analysis demonstrated a significant association between Type D personality and chronic NSLBP group status (Table 4). Individuals with Type D personality showed an increased likelihood of belonging to the NSLBP group compared with those without Type D personality (OR = 3.891, 95% CI: 1.169 to 12.954, *p* = 0.027). BMI was also associated with group membership (OR = 1.233, 95% CI: 1.053 to 1.444, *p* = 0.009). Multicollinearity was not considered a concern, as all variance inflation factor (VIF) values were below 2.

Multivariable linear regression analyses were performed within the NSLBP group to examine the adjusted associations of Type D personality with clinical, psychosocial, and quality-of-life outcomes (Table 5). After controlling for age, sex, BMI, and pain duration, Type D personality remained associated with increased VAS scores (B = 1.926, 95% CI: 0.660 to 3.191, *p* = 0.004). The VAS model was statistically significant and explained 45.8% of the variance in pain intensity scores *F*(5, 28) = 4.739, *p* = 0.003; R^2^ = 0.458; adjusted R^2^ = 0.362). For ODI scores, the overall adjusted model was not statistically significant *F*(5, 28) = 1.901, *p* = 0.126; R^2^ = 0.253; adjusted R^2^ = 0.120). Although Type D personality was significantly associated with higher ODI scores after adjustment for age, sex, BMI, and pain duration (B = 13.662, 95% CI: 3.985 to 23.340, *p* = 0.007), this finding should be interpreted cautiously given the non-significant overall model. In the adjusted model for SF-12 MCS, Type D personality was linked to poorer mental quality-of-life scores (B = −11.616, 95% CI: −19.060 to −4.174, *p* = 0.003). This model was statistically significant and explained 45.3% of the variance in SF-12 MCS scores *F*(5, 28) = 4.635, *p* = 0.003; R^2^ = 0.453; adjusted R^2^ = 0.355). In contrast, the adjusted model for SF-12 PCS was not statistically significant *F*(5, 28) = 0.231, *p* = 0.946; R^2^ = 0.040; adjusted R^2^ = −0.132), and no independent association was identified between Type D personality and SF-12 PCS scores (B = −1.303, 95% CI: −6.820 to 4.213, *p* = 0.632). For RSES scores, the adjusted model was not statistically significant *F*(5, 28) = 2.175, *p* = 0.086; R^2^ = 0.280; adjusted R^2^ = 0.151). After adjustment for age, sex, BMI, and pain duration, no significant relationship was observed between Type D personality and RSES scores (B = −1.532, 95% CI: −4.761 to 1.697, *p* = 0.339). Across all adjusted models, no evidence of multicollinearity was observed, and no influential observations were identified.

## 4. Discussion

Analysis of our data revealed that Type D personality traits are notably more prevalent in individuals with NSLBP than in healthy individuals. Within the patient group, it was associated with higher pain intensity, greater disability, increased anxiety and depressive symptoms, and lower mental quality-of-life scores. These associations were largely maintained after adjustment for age, sex, BMI, and pain duration, particularly for VAS, ODI, and SF-12 MCS scores. No significant relationship was found with SF-12 PCS or RSES in the adjusted models. Taken together, these findings suggest that Type D personality may be more closely related to the psychological and functional aspects of chronic NSLBP. However, due to the cross-sectional design, causal inferences cannot be made.

Our findings, revealing a 50% prevalence of Type D personality in the NSLBP group, align with previous literature highlighting a heightened frequency of this trait in chronic pain syndromes. Specifically, the prevalence observed in our cohort resonates with findings in fibromyalgia and myofascial pain syndrome, where Type D traits reportedly fluctuate between 30% and 64% [11,28,29,30,31]. A recent study in a university spine outpatient clinic reported a Type D personality prevalence of 32.3% and found that this personality profile was associated with greater pain, higher disability, and increased psychological distress [12]. Similarly, studies in fibromyalgia, temporomandibular disorders, and knee osteoarthritis have reported associations between Type D personality and poorer patient-reported outcomes, including psychological distress and quality-of-life measures [30,32,33]. Taken together, these findings support the relevance of Type D personality as a psychosocial correlate in chronic musculoskeletal pain conditions, while emphasizing the need for cautious interpretation regarding causality and specificity.

When patients with chronic NSLBP were evaluated according to Type D personality status, those with Type D personality had higher pain intensity, greater disability, higher anxiety and depressive symptom scores, and lower SF-12 MCS scores. Age, BMI, and pain duration did not differ significantly between Type D-positive and Type D-negative patients. These subgroup findings were supported in part by the adjusted regression analyses, in which Type D personality was associated with VAS, ODI, and SF-12 MCS scores after adjustment for age, sex, BMI, and pain duration. In contrast, Type D personality was not associated with SF-12 PCS or RSES scores in the adjusted models. Previous research in chronic low back pain has similarly suggested that personality-related characteristics may be associated with pain-related cognitive and emotional responses, such as fear of movement and pain catastrophizing, as well as pain and disability [13]. Therefore, personality-related factors may represent clinically relevant psychosocial correlates of pain-related burden in chronic NSLBP.

In a study conducted in patients with fibromyalgia and healthy controls, no significant difference in self-esteem levels was observed between the groups [31]. Similarly, we found no significant difference between patients with chronic NSLBP and healthy controls, and RSES scores did not differ significantly according to Type D personality status within the patient group. In the adjusted regression analysis, Type D personality was also not associated with RSES scores. However, exploratory correlation analyses showed that lower self-esteem was correlated with higher Type D-related scores and higher anxiety and depressive symptom scores. These findings suggest that self-esteem may be more closely related to broader psychological distress and personality-related traits than to NSLBP group status in this sample. Nevertheless, given the cross-sectional design and exploratory nature of the correlation analyses, these observations should be interpreted cautiously.

Type D personality has been most extensively studied in cardiovascular populations, where it has been associated with poorer quality of life and adverse clinical outcomes [8,34,35]. Although findings from cardiovascular research cannot be directly extrapolated to chronic NSLBP, they support the broader relevance of Type D personality as a psychosocial construct associated with patient-reported outcomes across chronic conditions.

The mechanisms linking Type D personality with pain intensity, disability, and mental quality of life in chronic NSLBP are not fully understood. Several psychological, behavioral, and biological pathways may be relevant. The negative affectivity component of Type D personality overlaps conceptually with anxiety and depressive symptoms, while social inhibition may be related to reduced emotional expression, lower perceived social support, and less adaptive coping responses. Previous studies have suggested that individuals with Type D personality may be more likely to use passive or avoidant coping strategies and less likely to engage in health-promoting behaviors [36,37]. In spine outpatient populations, Type D personality has been associated with greater pain, disability, and psychological distress [12], and stress-related autonomic, neuroendocrine, inflammatory, or endothelial pathways have also been proposed as possible mechanisms linking Type D personality with psychological distress [38]. However, these mechanisms were not directly tested in the present study and should therefore be considered.

An important issue is the specificity of Type D personality in relation to broader psychological distress. The NA component of Type D personality may overlap conceptually and statistically with anxiety and depressive symptoms measured by HADS. Because of the modest sample size and the risk of overfitting, HADS-A and HADS-D were not included in the primary adjusted regression models. Therefore, it remains uncertain whether the observed associations are specific to Type D personality itself or partly reflect a broader burden of psychological distress. This issue should be addressed in larger studies using models specifically designed to separate personality-related traits from anxiety, depression, catastrophizing, and fear-avoidance constructs.

Collectively, our findings support the relevance of Type D personality as a psychosocial factor associated with pain intensity, disability, psychological distress, and mental quality of life in chronic NSLBP. However, Type D personality should not be interpreted as a causal determinant of these outcomes based on the present cross-sectional data.

Certain limitations inherent to the present study warrant acknowledgment when interpreting the findings. First, the cross-sectional design does not allow causal or temporal interpretations regarding the relationships among Type D personality, psychological distress, pain intensity, disability, and quality of life. Second, the investigation’s single-center framework and relatively constrained cohort dimensions might impact the external validity and the overall sensitivity of the statistical inferences. Third, although adjusted regression models were performed, the possibility of residual confounding related to unmeasured variables, including occupational factors, physical activity, pain-related cognitions, coping strategies, and social support, cannot be ruled out. Fourth, the exclusion of individuals with known psychiatric disorders or regular psychiatric treatment may have narrowed the range of psychological distress and may restrict the generalizability of the findings to the wider chronic NSLBP population. Fifth, the control group was selected to have no history of chronic spinal pain or musculoskeletal disorders, which may have resulted in a particularly healthy comparison group and may have influenced between-group differences. Sixth, because of the modest sample size and the conceptual overlap between Type D personality, particularly negative affectivity, and anxiety/depressive symptoms, HADS-A and HADS-D were not included in the primary multivariable models to avoid overfitting and potential overadjustment. Accordingly, the extent to which Type D personality represents an independent construct beyond general psychological distress should be interpreted with caution. Finally, the use of self-reported outcome measures and the number of exploratory comparisons and correlations may increase the risk of response bias and type I error.

## 5. Conclusions

Type D personality characteristics were more frequent in patients with chronic NSLBP than in healthy controls and were associated with higher pain intensity, greater disability, higher psychological distress, and poorer mental quality-of-life scores within the patient group. In adjusted analyses, Type D personality was associated with VAS, ODI, and SF-12 MCS scores, but not with SF-12 PCS or RSES scores. Self-esteem did not differ significantly between patients and controls. These findings support the relevance of psychosocial assessment in chronic NSLBP; however, they should be interpreted as associations rather than causal effects because of the cross-sectional design and the conceptual overlap between negative affectivity and psychological distress. To fully elucidate the temporal trajectories and the predictive significance of Type D personality in chronic NSLBP, subsequent large-scale longitudinal investigations are warranted.

## Figures and Tables

**Table 1:** Comparison of Demographic and Clinical Characteristics Between the Study Groups.

Variable	Patient (*n* = 34)	Control (*n* = 34)	Effect Size	*p*-Value
Age, years	34.0 (25.5 to 44.5)	31.0 (28.0 to 42.8)	0.027 (−0.245 to 0.294)	0.854
BMI (kg/m^2^)	24.8 (22.9 to 29.5)	24.4 (22.0 to 27.4)	−0.226 (−0.467 to 0.047)	0.111
Sex				
Female	30 (88.2%)	27 (79.4%)	0.120	0.512
Male	4 (11.8%)	7 (20.6%)		
Marital Status				
Married	24 (70.6%)	19 (55.9%)	0.152	0.209
Single	10 (29.4%)	15 (44.1%)		
Comorbidities				
Diabetes mellitus	2 (5.9%)	0 (0.0%)	0.174	0.493
Hypertension	2 (5.9%)	0 (0.0%)	0.174	0.493
Asthma	2 (5.9%)	0 (0.0%)	0.174	0.493
Thyroid disease	5 (14.7%)	1 (2.9%)	0.207	0.197
Smoking	3 (8.8%)	9 (26.5%)	−0.231	0.109

Values are expressed as median (interquartile range) or *n* (%), depending on variable type. Group comparisons for continuous variables were performed using the Mann–Whitney U test, with rank-biserial correlation and corresponding 95% confidence intervals reported as effect size measures. Categorical variables were evaluated using the chi-square or Fisher’s exact test when appropriate, and effect sizes for 2 × 2 tables were presented as phi coefficients. Statistical significance was defined as *p* < 0.05. BMI: body mass index, *n*: number of participants.

**Table 2:** Patient-specific clinical characteristics and between-group comparison of psychosocial measures.

Variable	Patient (*n* = 34)	Control (*n* = 34)	Effect Size	*p*-Value
**Patient-specific clinical characteristics**				
VAS	7.0 ± 2.0	—	—	—
Pain duration, months	24 (12–81)	—	—	—
Oswestry Disability Index	24.5 (18.5 to 35.5)	—	—	—
**Between-group psychosocial and quality-of-life measures**				
Rosenberg Self-Esteem Scale	22.1 ± 4.5	23.3 ± 4.6	−0.270 (−0.747 to 0.208)	0.269
Type D personality (n/%)	17 (50.0%)	7 (20.6%)	0.308	0.011
Type D—Negative Affectivity	17.0 (9.0 to 20.8)	8.0 (4.3 to 12.0)	0.488 (0.251 to 0.669)	<0.001
Type D—Social Inhibition	10.5 (4.5 to 15.8)	8.0 (4.0 to 12.8)	0.158 (−0.116 to 0.41)	0.263
Type D composite score (NA × SI)	172.0 (70.5 to 329.8)	84.0 (26.3 to 157.3)	0.355 (0.094 to 0.57)	0.012
HADS—Depression	6.5 (3.3 to 9.0)	6.0 (4.0 to 8.0)	0.067 (−0.206 to 0.331)	0.635
HADS—Anxiety	9.0 (3.8 to 13.0)	5.0 (3.0 to 6.8)	0.378 (0.121 to 0.588)	0.007
SF-12 Physical Component Summary	37.3 (31.8 to 39.8)	53 (43.4 to 56.3)	−0.800 (−0.880 to −0.677)	<0.001
SF-12 Mental Component Summary	41.5 (36.7 to 54.3)	47.6 (42.6 to 54.7)	−0.116 (−0.374 to 0.159)	0.415

Data are shown as mean ± standard deviation, median (interquartile range), or *n* (%), depending on variable characteristics. Clinical variables specific to patients were reported only for the chronic NSLBP group and were not used in groupwise comparisons. Type D personality classification was treated as the main categorical Type D variable, whereas NA, SI, and NA × SI scores were evaluated as secondary dimensional measures. Depending on data distribution, continuous variables were analyzed using Student’s *t*-test or the Mann–Whitney U test. Effect size measures included Cohen’s d for parametric analyses and rank-biserial correlation with 95% confidence intervals for nonparametric comparisons. Categorical variables were examined using chi-square or Fisher’s exact tests, and phi coefficients were reported for 2 × 2 contingency tables. Statistical significance was defined as *p* < 0.05. VAS: Visual Analog Scale; HADS: Hospital Anxiety and Depression Scale; NA: negative affectivity; SI: social inhibition; SF-12: 12-Item Short Form Health Survey, *n*: number of participants, —: not applicable; these variables were assessed only in the patient group, and no between-group comparison was performed.

**Table 3:** Exploratory comparison of clinical and psychosocial characteristics of patients with chronic nonspecific low back pain according to Type D personality status.

Variable	Type D Personality (−)	Type D Personality (+)	Effect Size	*p*-Value
Age, years	39 (28 to 45)	29 (20 to 36)	−0.329 (−0.626 to 0.052)	0.105
BMI (kg/m^2^)	26.8 (23.5 to 31.3)	24.2 (22.8 to 27.9)	−0.263 (−0.58 to 0.124)	0.196
VAS	6 (5 to 7)	8 (7 to 10)	0.592 (0.279 to 0.791)	0.003
Pain duration, months	30 (12 to 84)	24 (12 to 36)	−0.121 (−0.474 to 0.266)	0.555
Oswestry Disability Index	20 (16 to 26)	30 (22 to 42)	0.498 (0.152 to 0.736)	0.014
Rosenberg Self-Esteem Scale	25 (20 to 26)	19 (18 to 25)	−0.353 (−0.643 to 0.025)	0.080
HADS—Depression	4 (2 to 7)	8 (6 to 9)	0.453 (0.095 to 0.708)	0.025
HADS—Anxiety	3 (1 to 6)	13 (9 to 14)	0.817 (0.637 to 0.912)	<0.001
SF-12 Physical Component Summary	37.7 (35.1 to 41.3)	36.9 (29.5 to 38.9)	−0.149 (−0.496 to 0.239)	0.474
SF-12 Mental Component Summary	53.20 (40.3 to 59.0)	38.9 (33.4 to 42.2)	−0.606 (−0.799 to −0.299)	0.002

Values are expressed as median (interquartile range). Comparisons according to Type D personality status within the patient group were conducted as exploratory subgroup analyses. Continuous variables were analyzed using the Mann–Whitney U test, and effect sizes are presented as rank-biserial correlations with corresponding 95% confidence intervals. BMI: body mass index; VAS: Visual Analog Scale; HADS: Hospital Anxiety and Depression Scale; SF-12: 12-Item Short Form Health Survey.

**Table 4:** Adjusted binary logistic regression analysis for chronic nonspecific low back pain group membership.

Variable	B ± SE	OR (95% CI)	*p*-Value	Tolerance	VIF
Age	−0.044 ± 0.033	0.957 (0.898 to 1.021)	0.186	0.641	1.561
Sex, male	−1.480 ± 1.027	0.228 (0.030 to 1.704)	0.150	0.738	1.355
BMI	0.210 ± 0.081	1.233 (1.053 to 1.444)	0.009	0.611	1.638
Type D personality, present	1.359 ± 0.614	3.891 (1.169 to 12.954)	0.027	0.946	1.057

Model fit: χ^2^ = 16.004, *p* = 0.003; Nagelkerke R^2^ = 0.280; AUC = 0.773. In the logistic regression model, group classification was coded as 0 = healthy control and 1 = chronic nonspecific low back pain. The reference categories were female for sex and absence of Type D personality for Type D status. B: unstandardized regression coefficient; OR: odds ratio; CI: confidence interval; SE: standard error; BMI: body mass index; VIF: variance inflation factor.

**Table 5:** Adjusted linear regression analyses for clinical, psychosocial, and quality-of-life outcomes in patients with chronic NSLBP.

Predictor	B (95% CI)	*p*-Value
Outcome: VAS		
Age	−0.085 (−0.153 to −0.018)	0.015
Sex, male	−0.932 (−2.769 to 0.905)	0.308
BMI	0.116 (−0.009 to 0.240)	0.067
Pain duration	0.006 (−0.003 to 0.014)	0.188
Type D personality, present	1.926 (0.660 to 3.191)	0.004
Outcome: ODI		
Age	−0.013 (−0.529 to 0.503)	0.958
Sex, male	4.925 (−9.125 to 18.980)	0.479
BMI	0.040 (−0.909 to 0.990)	0.931
Pain duration	0.043 (−0.021 to 0.108)	0.182
Type D personality, present	13.662 (3.985 to 23.340)	0.007
Outcome: SF-12 MCS		
Age	0.212 (−0.184 to 0.609)	0.282
Sex, male	4.792 (−6.014 to 15.600)	0.371
BMI	−0.836 (−1.566 to −0.106)	0.026
Pain duration	0.041 (−0.008 to 0.091)	0.098
Type D personality, present	−11.616 (−19.060 to −4.174)	0.003
Outcome: SF-12 PCS		
Age	−0.014 (−0.308 to 0.280)	0.923
Sex, male	0.680 (−7.330 to 8.689)	0.863
BMI	0.026 (−0.515 to 0.568)	0.921
Pain duration	−0.018 (−0.054 to 0.019)	0.338
Type D personality, present	−1.303 (−6.820 to 4.213)	0.632
Outcome: RSES		
Age	0.218 (0.046 to 0.390)	0.015
Sex, male	0.398 (−4.290 to 5.086)	0.863
BMI	−0.227 (−0.544 to 0.090)	0.153
Pain duration	0.009 (−0.013 to 0.031)	0.399
Type D personality, present	−1.532 (−4.761 to 1.697)	0.339

Separate multivariable linear regression models were constructed within the chronic NSLBP group. Each model included age, sex, BMI, pain duration, and Type D personality status as predictor variables. Results are presented as unstandardized regression coefficients with corresponding 95% confidence intervals. Female sex and absence of Type D personality were used as reference categories. Model fit statistics were as follows: VAS model, *F*(5, 28) = 4.739, *p* = 0.003, R^2^ = 0.458, adjusted R^2^ = 0.362; ODI model, *F*(5, 28) = 1.901, *p* = 0.126, R^2^ = 0.253, adjusted R^2^ = 0.120; SF-12 MCS model, *F*(5, 28) = 4.635, *p* = 0.003, R^2^ = 0.453, adjusted R^2^ = 0.355; SF-12 PCS model, *F*(5, 28) = 0.231, *p* = 0.946, R^2^ = 0.040, adjusted R^2^ = −0.132; RSES model, *F*(5, 28) = 2.175, *p* = 0.086, R^2^ = 0.280, adjusted R^2^ = 0.151. All variance inflation factor (VIF) values were <2, and no influential cases were detected. B: unstandardized regression coefficient; BMI: body mass index; CI: confidence interval; MCS: Mental Component Summary; NSLBP: nonspecific low back pain; ODI: Oswestry Disability Index; PCS: Physical Component Summary; RSES: Rosenberg Self-Esteem Scale; SF-12: 12-Item Short Form Health Survey; VAS: Visual Analog Scale.

## Data Availability

The raw data supporting the conclusions of this article will be made available by the authors on request.

## Abbreviations

The main abbreviations used in the present study are provided below:

NSLBP	Nonspecific Low Back Pain
VAS	Visual Analog Scale
BMI	Body Mass Index
DS14	Type D Scale-14
NA	Negative Affectivity
SI	Social Inhibition
NA x SI	Type D composite score
RSES	Rosenberg Self-Esteem Scale
HADS	Hospital Anxiety and Depression Scale
SF-12	12-Item Short Form Health Survey
PCS	Physical Component Summary
MCS	Mental Component Summary
